# P12 Can, and should, we improve our metrics for quantifying AMR?

**DOI:** 10.1093/jacamr/dlae217.016

**Published:** 2025-01-23

**Authors:** R W Humphry, J C Evans, M K Henry, G J Gunn, S C Tongue, G T Innocent

**Affiliations:** SRUC, Centre for Epidemiology & Planetary Health, RAVIC, 9 Inverness Campus, IV2 5NA, Scotland; SRUC, Centre for Epidemiology & Planetary Health, RAVIC, 9 Inverness Campus, IV2 5NA, Scotland; SRUC, Centre for Epidemiology & Planetary Health, RAVIC, 9 Inverness Campus, IV2 5NA, Scotland; SRUC, Centre for Epidemiology & Planetary Health, RAVIC, 9 Inverness Campus, IV2 5NA, Scotland; SRUC, Centre for Epidemiology & Planetary Health, RAVIC, 9 Inverness Campus, IV2 5NA, Scotland; BioSS, Biomathematics & Statistics Scotland, JCMB, The King's Buildings, Peter Guthrie Tait Road, Edinburgh, EH9 3FD, Scotland

## Abstract

**Background:**

The metrics we use to quantify our world affect how we perceive it. This applies to antimicrobial resistance (AMR) and here we present a chain of work that demonstrates, for example, how the choice of laboratory method can affect estimates of prevalence of phenotypic AMR. In peer-reviewed publications it appears that the most commonly used metrics to describe AMR are based on the laboratory methods based on individual bacteria isolated from clinical infections rather than populations of bacteria. We propose a method that accounts for heterogeneity within a single-species bacterial population and suggest that taking a population approach more often could be of benefit to policymakers and practitioners.

**Objectives:**

We seek to present the variety of different methods available, their relative complexity, the resources required, and the amount of information they offer about AMR in bacterial populations.

**Methods:**

The latest method we have developed, and which we now propose, is called Quantitative Estimation of Population AMR (QEPA). It combines traditional laboratory phenotypic methods with recently available Bayesian statistical methods. Two key features of this method are (i) that it makes use of bacterial count information that would otherwise be discarded by traditional laboratory approaches, and (ii) that it allows for, and describes, variation in resistance to the antimicrobial of interest between different individual bacteria within a population of bacteria of the same species. We have run a validation experiment to test this method against artificially constructed bacterial (*Escherichia coli*) populations of known (controlled) AMR profile. QEPA was also deployed on fresh samples from a livestock (dairy farm) setting.

**Results:**

Validation experiments demonstrate that this method behaves as designed. QEPA estimates sample bacterial density and the percentage of the whole population that are resistant to the antimicrobial of interest. Experiments on fresh samples demonstrate a level of quantitative discrimination not normally provided by conventional methods.

**Conclusions:**

It remains to be demonstrated as to how more informative methods for quantitatively describing AMR could improve our prediction of transfer of resistance or of clinical outcomes following the administration of antimicrobials. Considering the need to preserve the efficacy of available antimicrobials for as long as possible, and the consequent need to use antimicrobial chemotherapy only where appropriate, QEPA might, alongside other AMR metrics and clinical evidence, inform decision making around the risks versus benefits of applying treatment in cases of clinical bacterial disease. Any improvements in predicting the transfer of resistance will have important policy implications. Improvements in predicting clinical outcomes will help practitioners. Finally, we consider how future research might be designed in order to quantify the benefits of using more informative metrics when measuring AMR.

